# Supplementary material for: Branched ubiquitin chain binding and deubiquitination by UCH37 facilitate proteasome clearance of stress-induced inclusions
Source: eLife. 2021 Nov 11;10:e72798. doi: 10.7554/eLife.72798 (PMC8635973; doi:10.7554/eLife.72798)
Supplement: Figure 1—source data 1. [file elife-72798-fig1-data1.docx]

Source data for Figure 1A, 1B and 1D. Cropped regions are shown by boxes.

Figure 1A_Gel #1 and Figure 1B_Gel #1

20

50

Mw

(kDa)


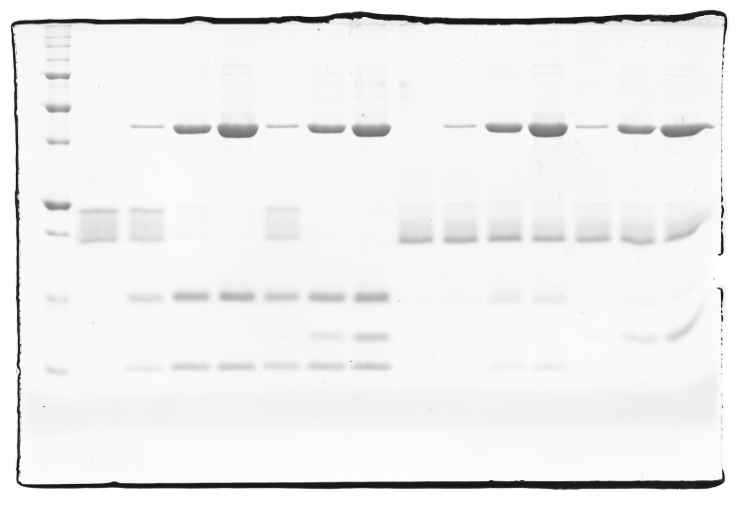


15

75

37

25

10

Figure 1A_Gel #2 and Figure 1A_Gel #3

Mw

(kDa)


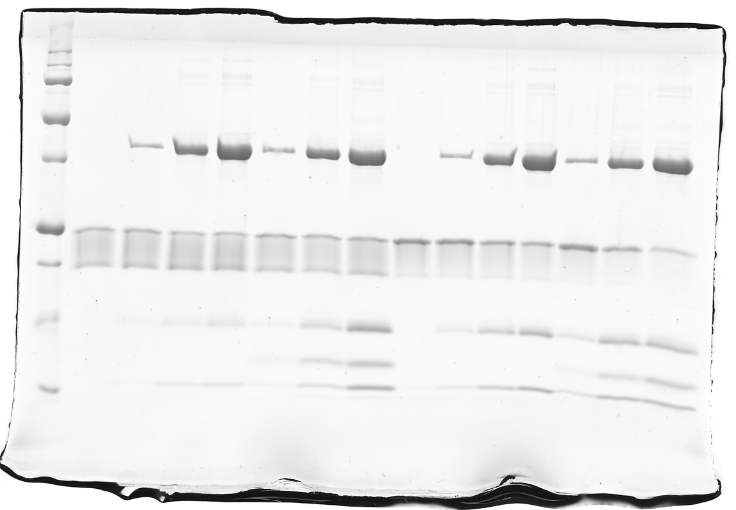


15

10

50

37

75

20

25

Figure 1B_Gel #2 and Figure 1B_Gel #3


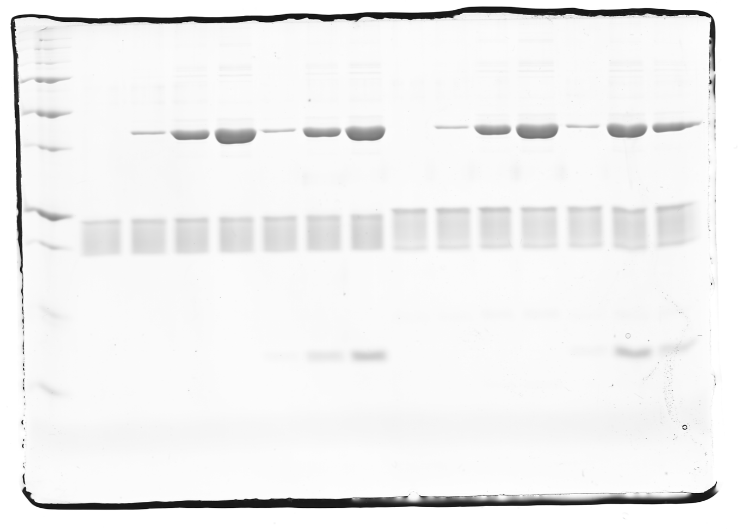


Mw

(kDa)

15

10

37

50

75

20

25

Figure 1B_ Gel #4


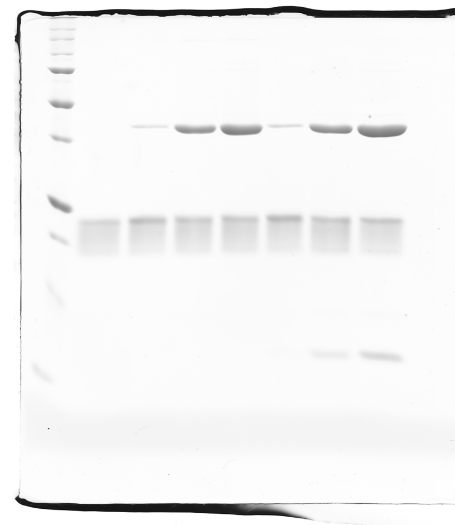


Mw

(kDa)

15

10

37

75

20

25

50

Figure 1D_Gel #1

Mw

(kDa)


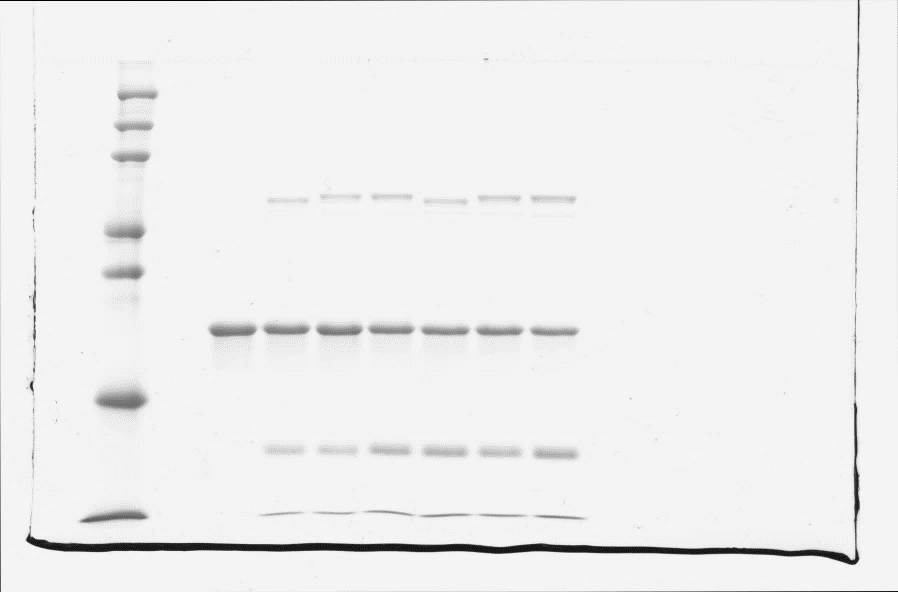


10

20

40

120

80

60

30

Replicate for Figure 1D_Gel #1 (Not shown in manuscript)

Mw

(kDa)

60

80


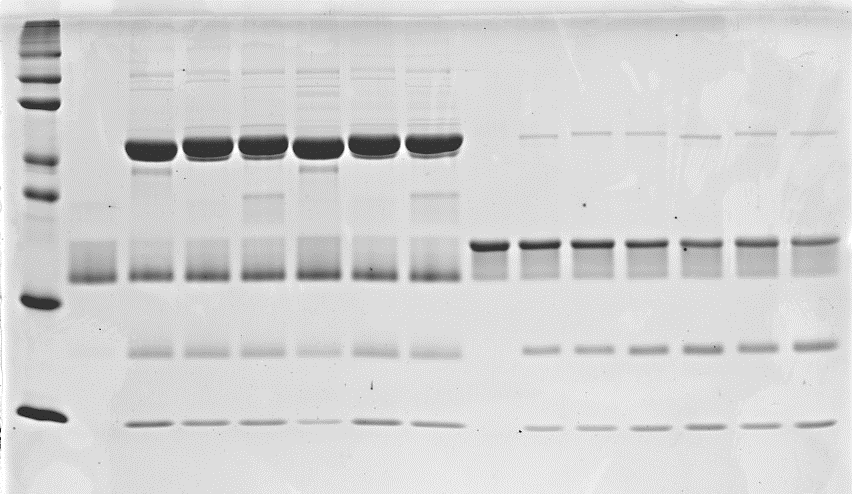


10

20

40

120

30

Figure 1D_Gel #2


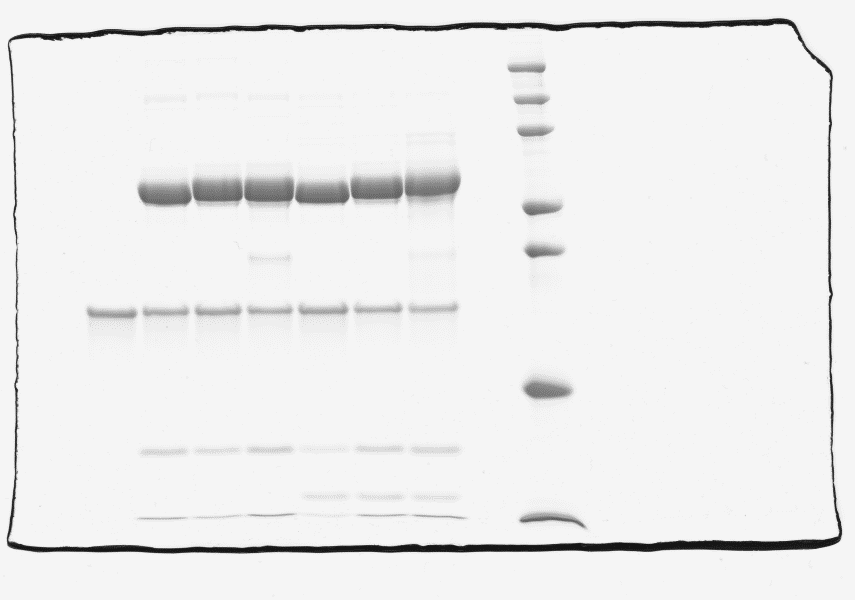


Mw

(kDa)

10

20

40

120

80

60

30

Replicate for Figure 1D_Gel #2 (Not shown in manuscript)


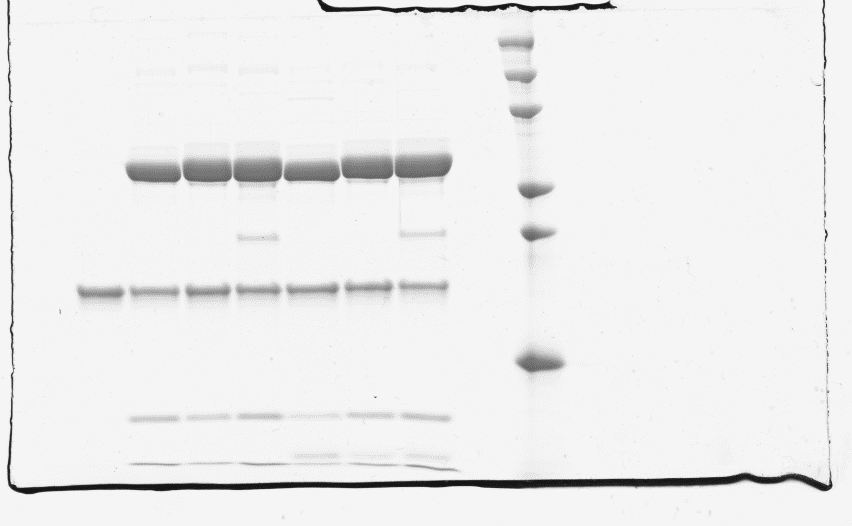


Mw

(kDa)

10

20

40

120

60

30

80
