# Supplementary figures and images for: Branched ubiquitin chain binding and deubiquitination by UCH37 facilitate proteasome clearance of stress-induced inclusions
Source: eLife. 2021 Nov 11;10:e72798. doi: 10.7554/eLife.72798 (PMC8635973; doi:10.7554/eLife.72798)

Source data for Figure 1-figure supplement 1 (B)

“Cropped regions are shown by boxes”


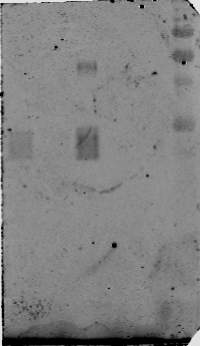

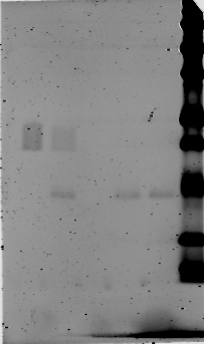


Blot: K48 (Green)

Supplement: Figure 1—figure supplement 1—source data 1. [file elife-72798-fig1-figsupp1-data1.docx]

Source data for Figure 3E. Cropped regions are shown by boxes.


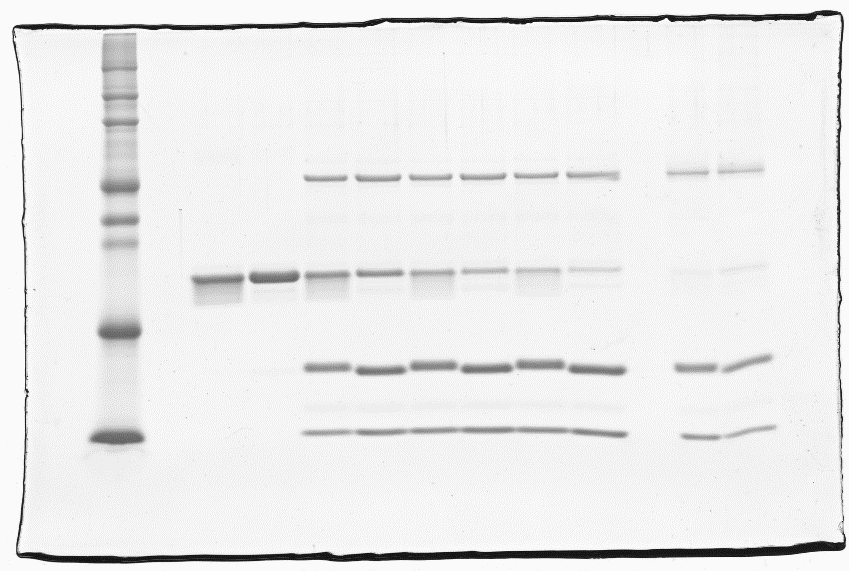

Supplement: Figure 3—source data 1. [file elife-72798-fig3-data1.docx]
