# Supplementary material for: Branched ubiquitin chain binding and deubiquitination by UCH37 facilitate proteasome clearance of stress-induced inclusions
Source: eLife. 2021 Nov 11;10:e72798. doi: 10.7554/eLife.72798 (PMC8635973; doi:10.7554/eLife.72798)

Source data for Figure 2-figure supplement 1(C)

“Cropped regions are shown by boxes”

Figure 2-figure supplement 1(C) Gel #1


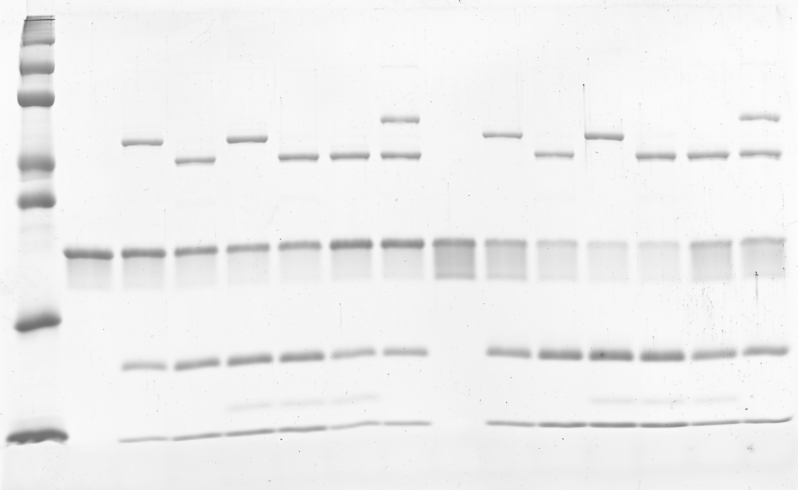


Figure 2-figure supplement 1(C) Gel #2


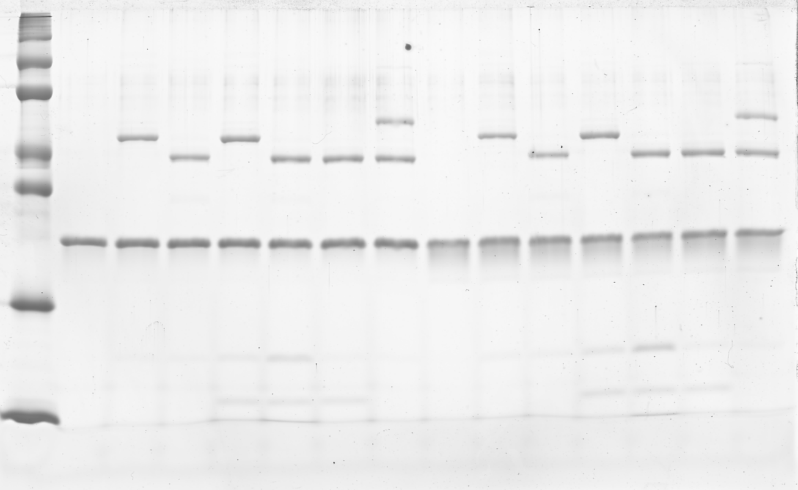


Figure 2-figure supplement 1(C) Gel #3


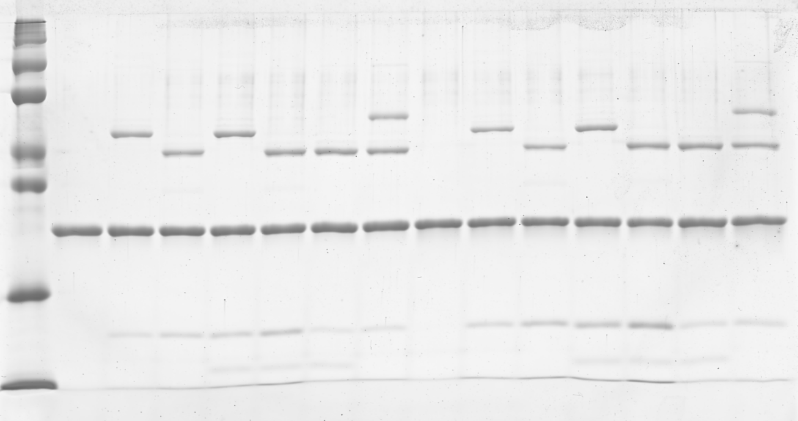

Supplement: Figure 2—figure supplement 1—source data 1. [file elife-72798-fig2-figsupp1-data1.docx]
