# Supplementary material for: Branched ubiquitin chain binding and deubiquitination by UCH37 facilitate proteasome clearance of stress-induced inclusions
Source: eLife. 2021 Nov 11;10:e72798. doi: 10.7554/eLife.72798 (PMC8635973; doi:10.7554/eLife.72798)

Source data for Figure 4 and the cropped regions are shown by boxes

Source data for Figure 4A


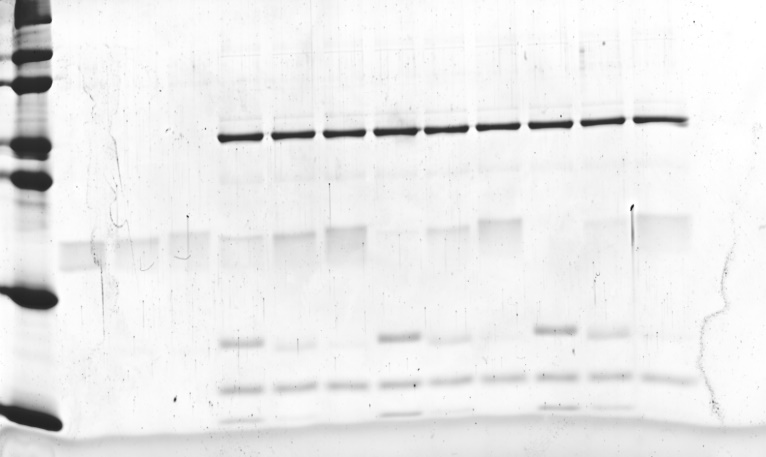


Source data for Figure 4C


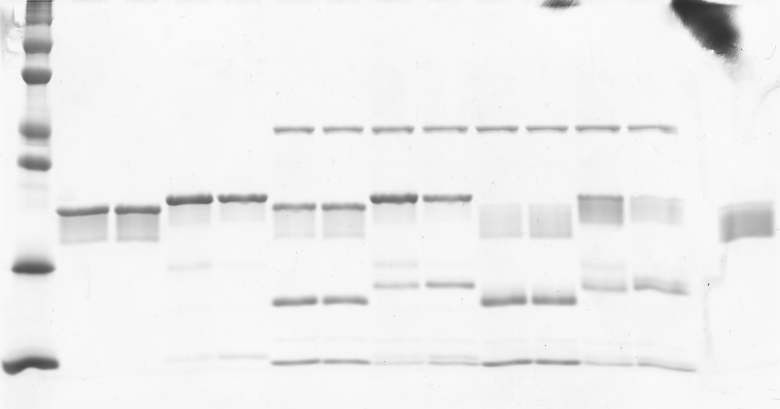


Source data for Figure 4D


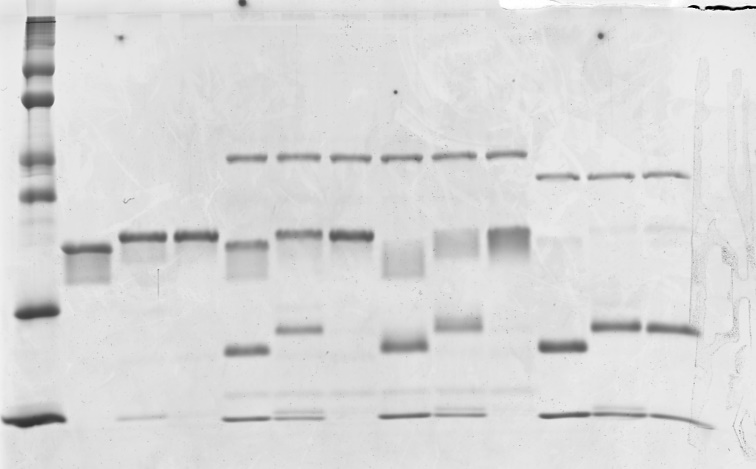

Supplement: Figure 4—source data 1. [file elife-72798-fig4-data1.docx]
