# Supplementary material for: Branched ubiquitin chain binding and deubiquitination by UCH37 facilitate proteasome clearance of stress-induced inclusions
Source: eLife. 2021 Nov 11;10:e72798. doi: 10.7554/eLife.72798 (PMC8635973; doi:10.7554/eLife.72798)
Supplement: Figure 5—source data 1. [file elife-72798-fig5-data1.docx]

Source data for Figure 5E and 5F. Cropped regions are shown by boxes.

Source data for Figure 5E and 5F.


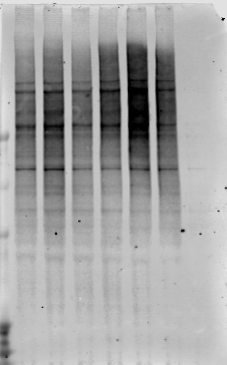

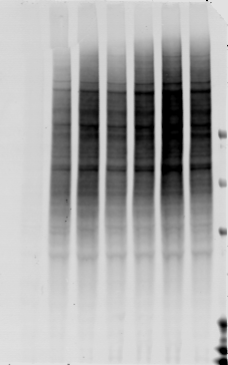


Blot: K11/48

Blot: K48


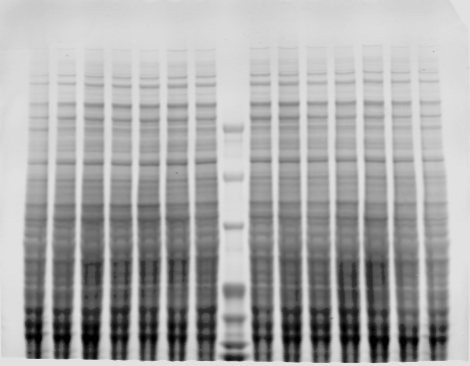


Total protein staining prior to K48 (left) or K11/48 blot (right)

101520 total protein

Source data for Figure 5F.


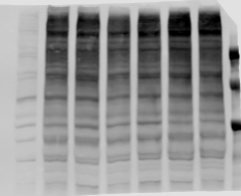

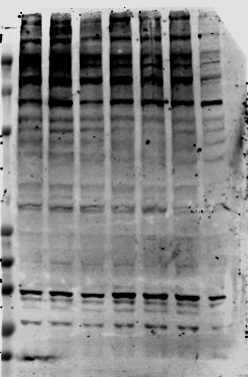

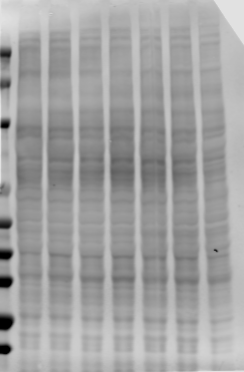


Blot: FK2

Blot: K63


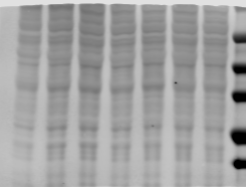


Total protein staining prior to FK2 blot

Total protein staining prior to K63 blot

Replicates for Figure 5F (not shown in manuscript).


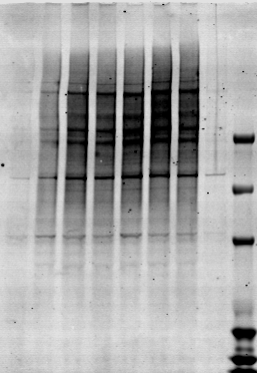

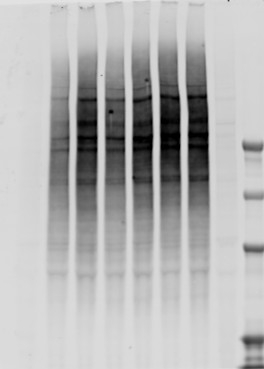

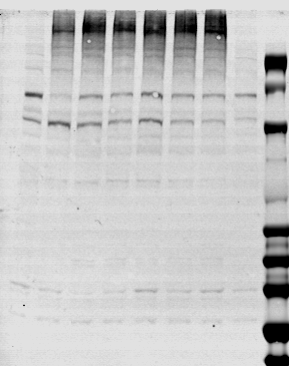


Blot: K63

Blot: K11/48

Blot: K48


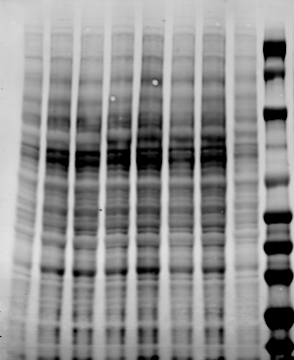

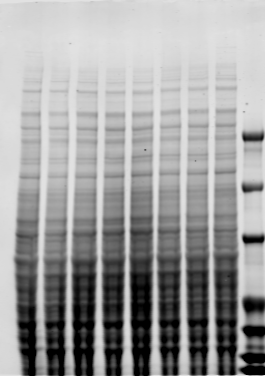

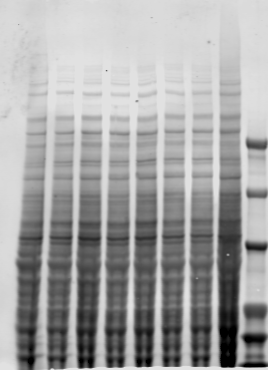


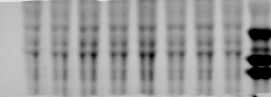


Total protein staining prior to K48 blot membrane

Total protein staining prior to K11/48 blot

Total protein staining prior to K63 blot


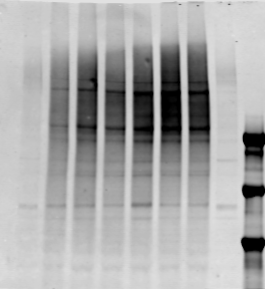


Total protein staining prior to FK2 blot

Blot: FK2
