# Supplementary material for: Branched ubiquitin chain binding and deubiquitination by UCH37 facilitate proteasome clearance of stress-induced inclusions
Source: eLife. 2021 Nov 11;10:e72798. doi: 10.7554/eLife.72798 (PMC8635973; doi:10.7554/eLife.72798)
Supplement: Figure 5—figure supplement 1—source data 1. [file elife-72798-fig5-figsupp1-data1.docx]

Source data for Figure 5-figure supplement 1B. Cropped regions are shown by boxes.


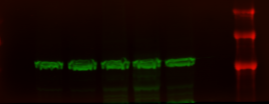

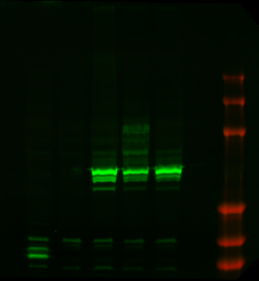

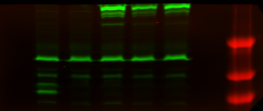

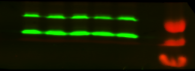


P KO WT C88A EWI

P KO WT C88A EWI

Blot: RPN2

*

Blot: Uch37(Rb)

Blot: Rpn13

Blot: PSMB5 (20S)
