# Supplementary material for: Branched ubiquitin chain binding and deubiquitination by UCH37 facilitate proteasome clearance of stress-induced inclusions
Source: eLife. 2021 Nov 11;10:e72798. doi: 10.7554/eLife.72798 (PMC8635973; doi:10.7554/eLife.72798)
Supplement: Figure 6—source data 1. [file elife-72798-fig6-data1.docx]

Source data for Figure 6A & 6B & 6D. Cropped regions are shown by boxes.

Source for figure 6A


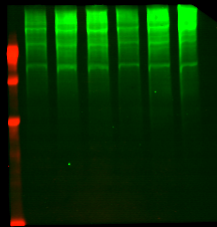

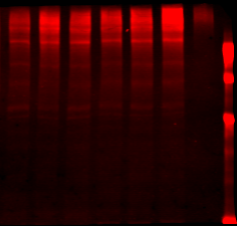


Blot: K11/48

Blot: FK2


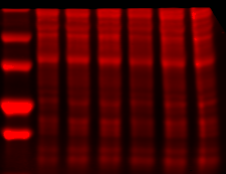

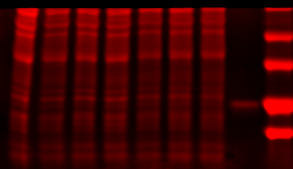


Total protein staining for FK2 blot

Total protein staining for K11/48 Ub blot


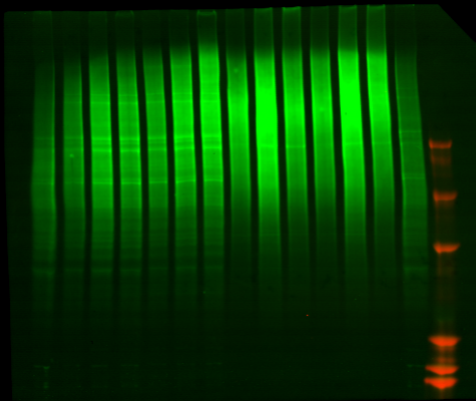


Blot: K48

Source data for Figure 6B


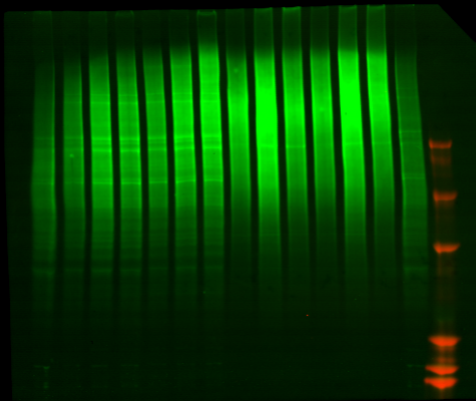

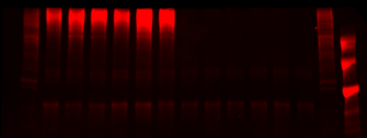


Blot: FK2


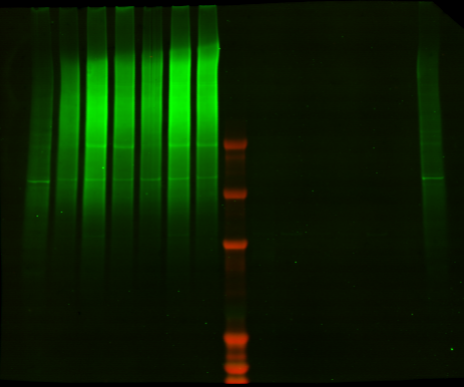


Blot: K48 Ub

Blot: K11/48 Ub


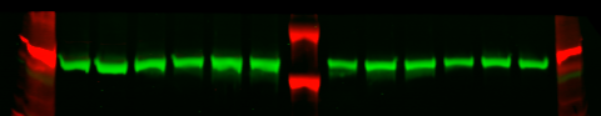


Blot: RPN2

Source data for Figure 6D

Input

Flag IP

Blot: Rad23B


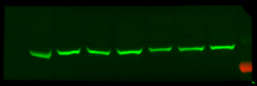

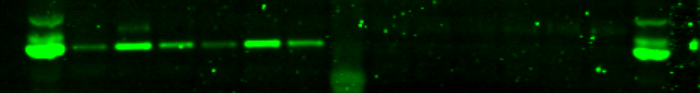


Blot: Rad23B
