# Supplementary material for: Branched ubiquitin chain binding and deubiquitination by UCH37 facilitate proteasome clearance of stress-induced inclusions
Source: eLife. 2021 Nov 11;10:e72798. doi: 10.7554/eLife.72798 (PMC8635973; doi:10.7554/eLife.72798)

Source data for Figure 6-figure supplement 1A. Cropped regions are shown by boxes.

Flag IP

Input


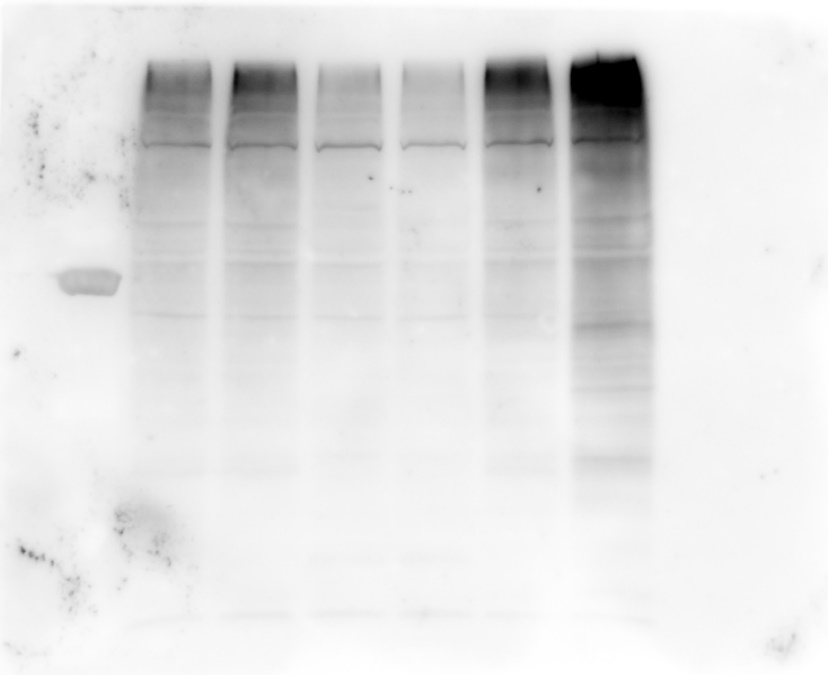

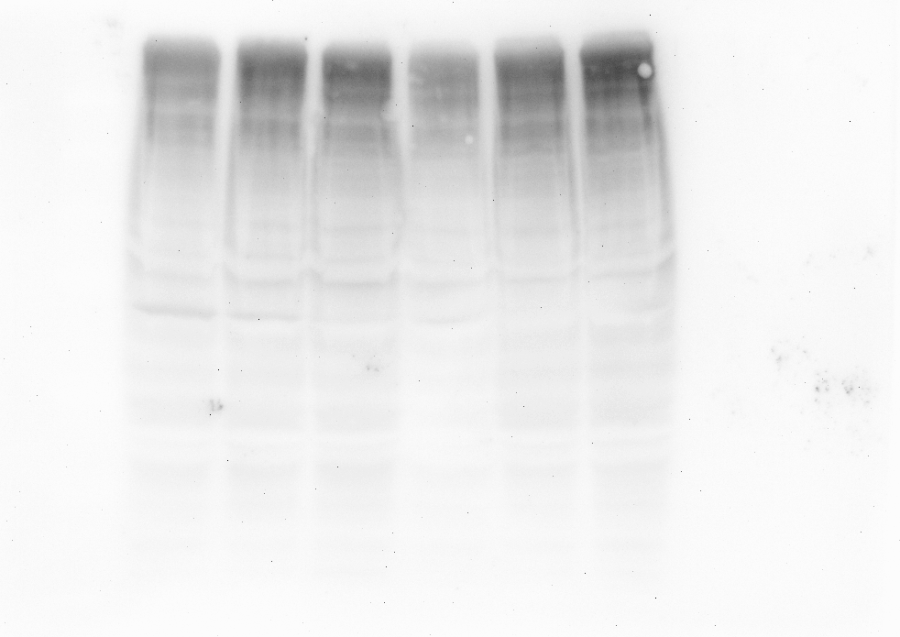

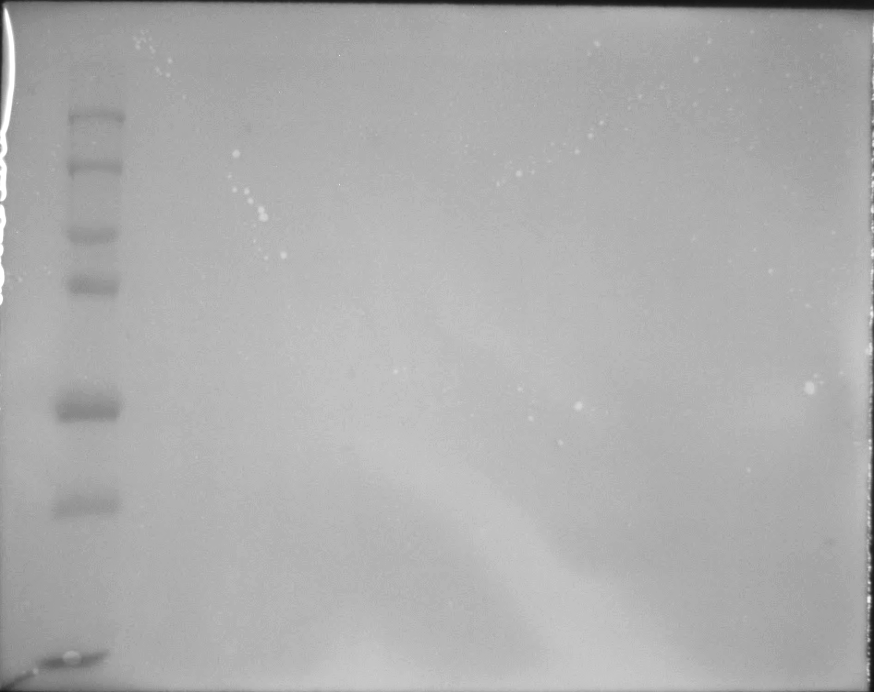


Blot: Ub


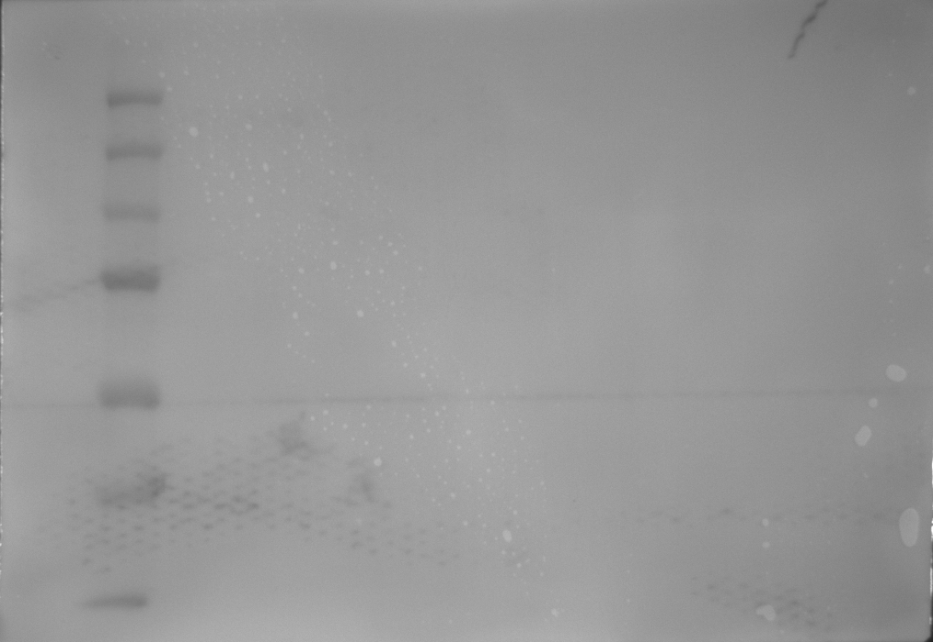


Blot: Ub


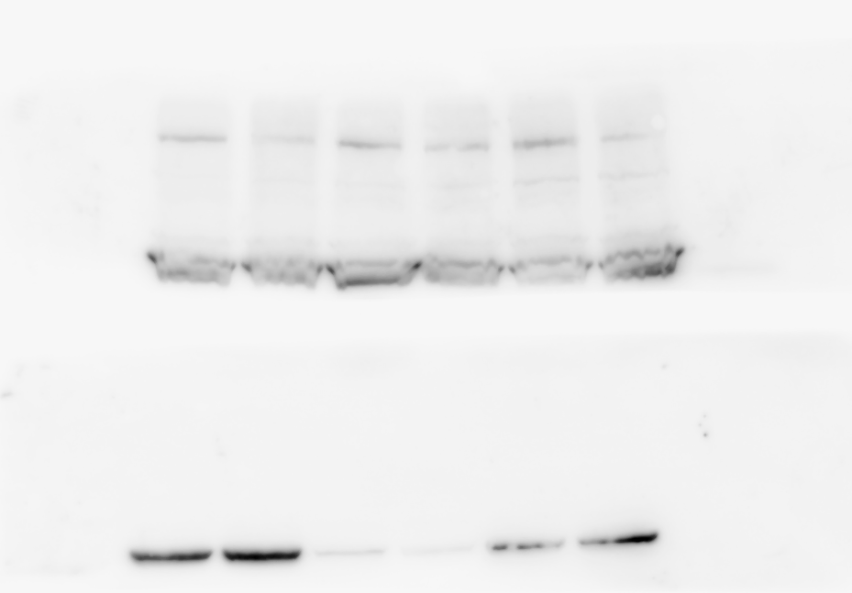

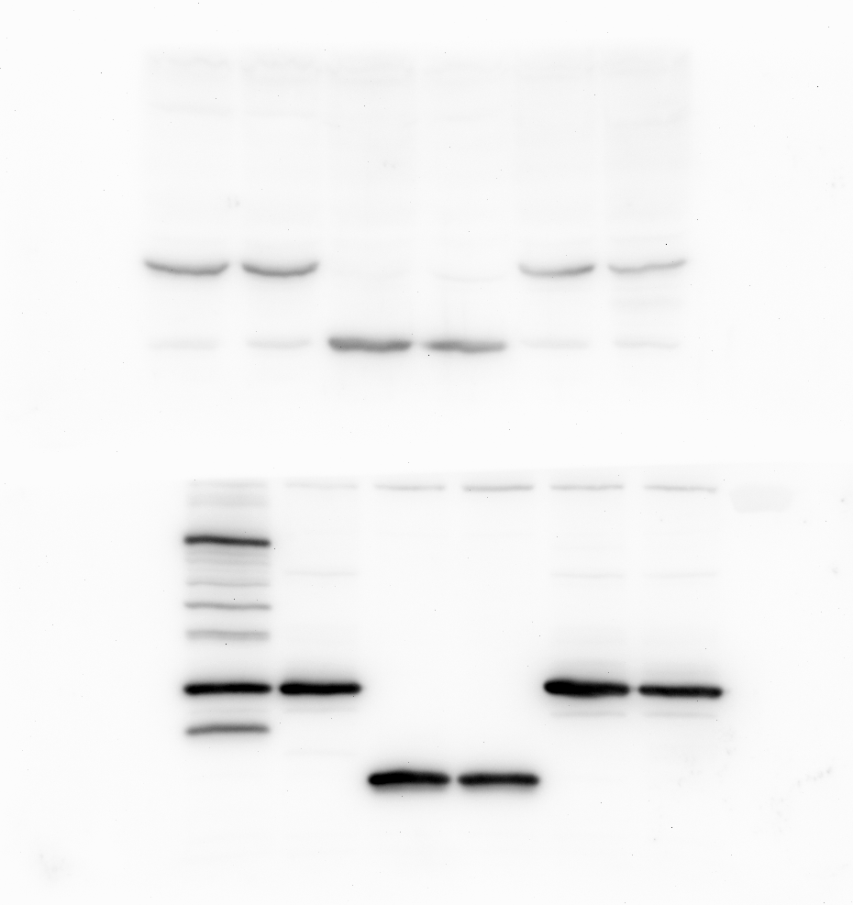

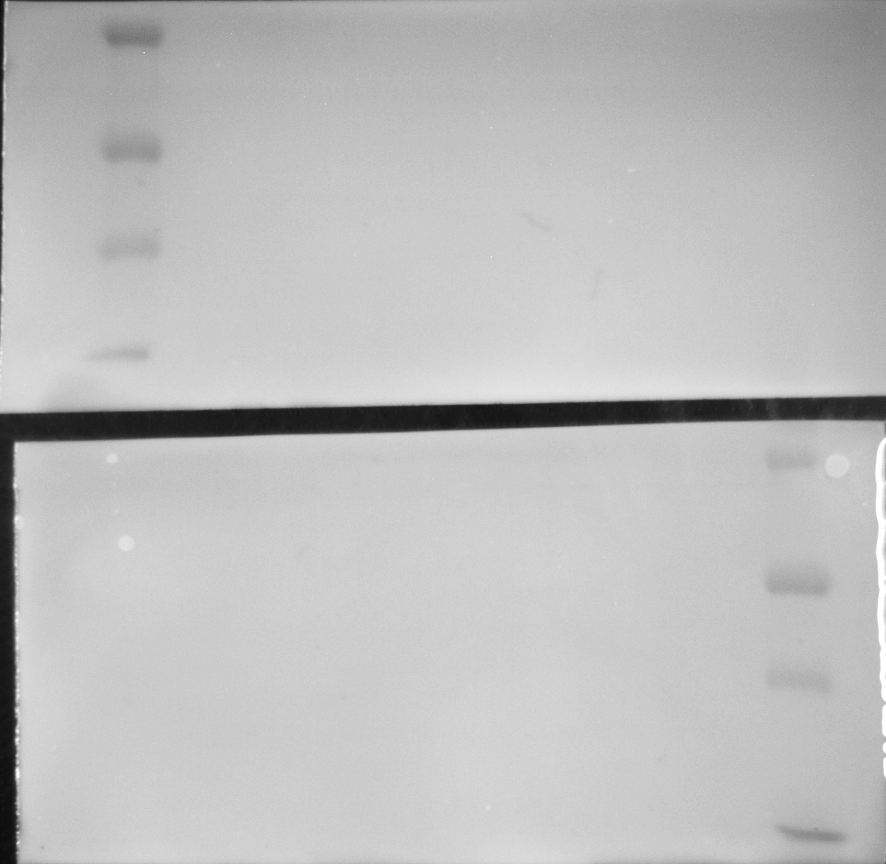


Input

Input


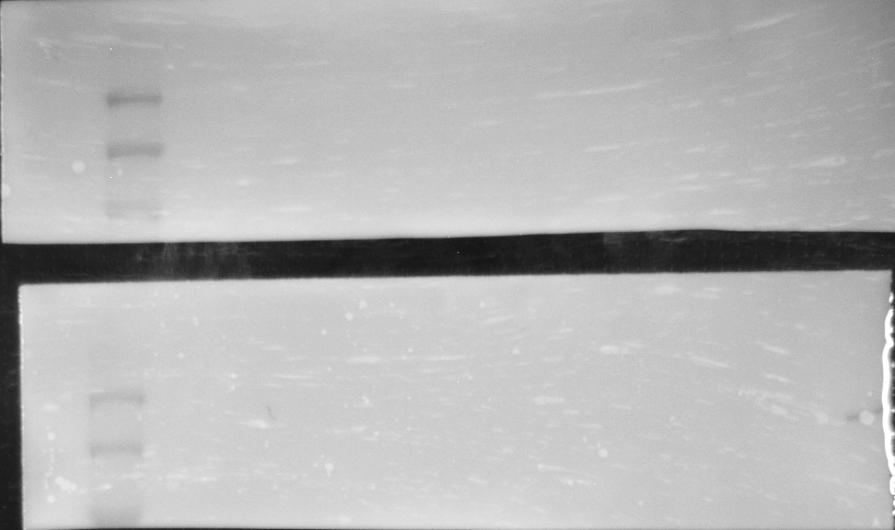


Flag IP

Flag IP

(flipped)

Blot: RPN2

Blot: Flag

Source data for Figure 6-figure supplement 1C. Cropped regions are shown by boxes.

Input Flag IP

Input Flag IP


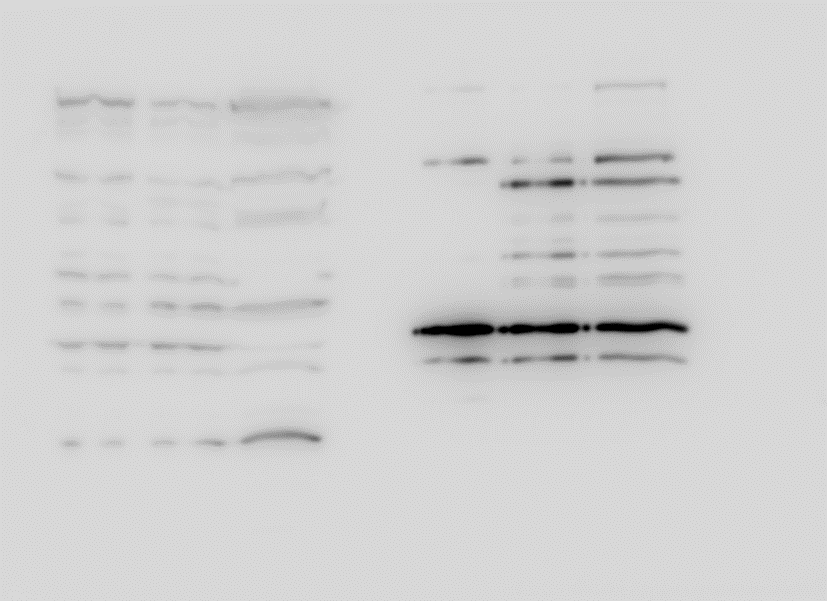


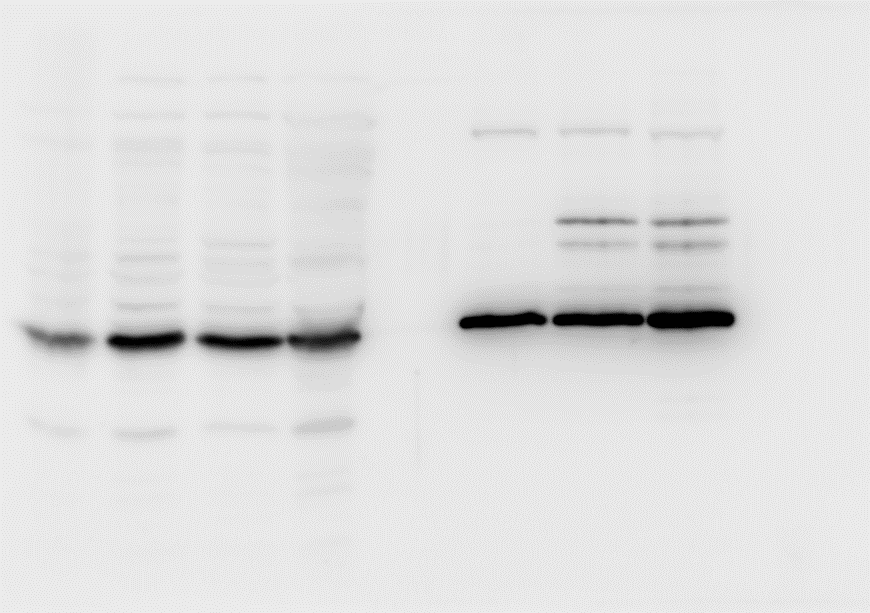

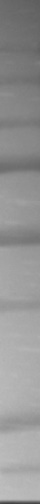

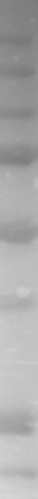


Blot: Flag

Blot: Rpn13


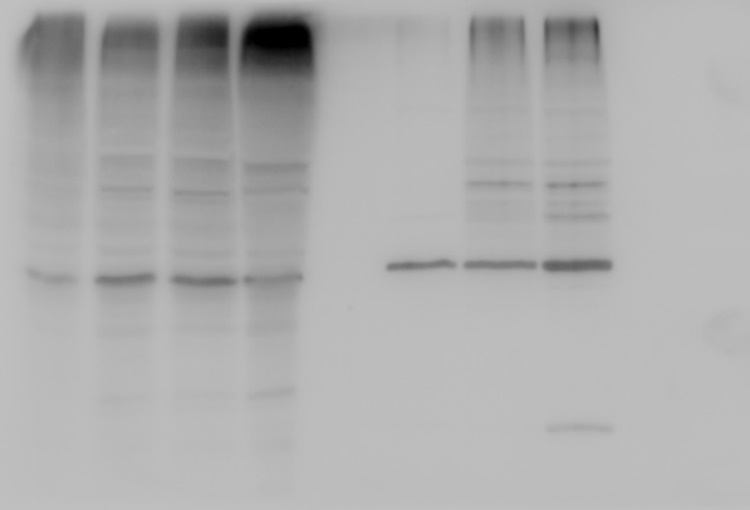

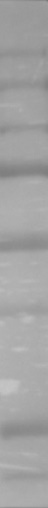


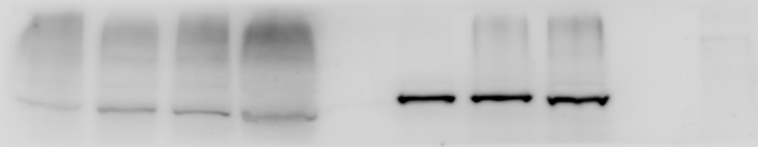

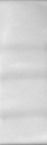


Blot: RPN2

Blot: Ub (After stripping from Rpn13 blot)

Source data for Figure 6-figure supplement 1B. Cropped regions are shown by boxes.


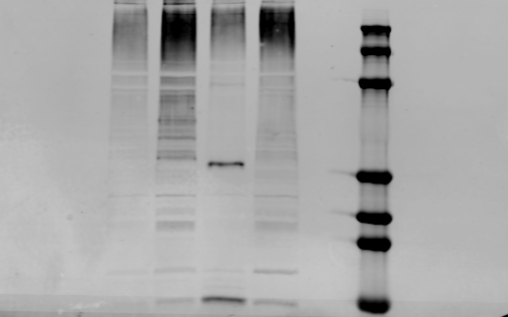


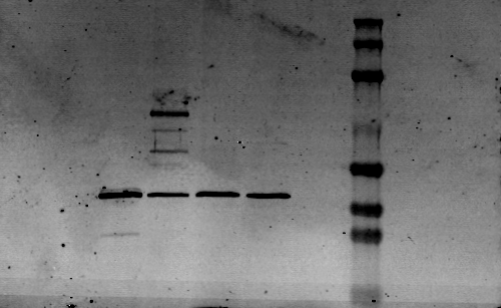


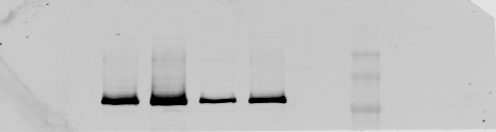

Supplement: Figure 6—figure supplement 1—source data 1. [file elife-72798-fig6-figsupp1-data1.docx]
