# Supplementary material for: Branched ubiquitin chain binding and deubiquitination by UCH37 facilitate proteasome clearance of stress-induced inclusions
Source: eLife. 2021 Nov 11;10:e72798. doi: 10.7554/eLife.72798 (PMC8635973; doi:10.7554/eLife.72798)
Supplement: Supplementary file 1. — The active site C88 is highlighted in yellow and EWI residues are indicated in orange. [file elife-72798-supp1.docx]

His-TEV-UCH37 MHHHHHHGKPIPNPLLGLDSTENLYFQGIDPFTMTGNAGEWCLMESDPGVFTELIKGFGC 60

NS-UCH37 ---------------------------------STGNAGEWCLMESDPGVFTELIKGFGC 27

UCH37deltaCTD ---------------------------------MTGNAGEWCLMESDPGVFTELIKGFGC 27

**************************

His-TEV-UCH37 RGAQVE**E**I**W**SLEPENFEKLKPVHGLIFLFKWQPGEEPAGSVVQDSRLDTIFFAKQVINNA 120

NS-UCH37 RGAQVE**E**I**W**SLEPENFEKLKPVHGLIFLFKWQPGEEPAGSVVQDSRLDTIFFAKQVINNA 87

UCH37deltaCTD RGAQVE**E**I**W**SLEPENFEKLKPVHGLIFLFKWQPGEEPAGSVVQDSRLDTIFFAKQVINNA 87

************************************************************

His-TEV-UCH37 **C**ATQAIVSVLLNCTHQDVHLGETLSEFKEFSQSFDAAMKGLALSNSDVIRQVHNSFARQQ 180

NS-UCH37 **C**ATQAIVSVLLNCTHQDVHLGETLSEFKEFSQSFDAAMKGLALSNSDVIRQVHNSFARQQ 147

UCH37deltaCTD **C**ATQAIVSVLLNCTHQDVHLGETLSEFKEFSQSFDAAMKGLALSNSDVIRQVHNSFARQQ 147

************************************************************

His-TEV-UCH37 MFEFDTKTSAKEEDAFHFVSYVPVNGRLYELDGLREGPIDLGACNQDDWISAVRPVIEKR 240

NS-UCH37 MFEFDTKTSAKEEDAFHFVSYVPVNGRLYELDGLREGPIDLGACNQDDWISAVRPVIEKR 207

UCH37deltaCTD MFEFDTKTSAKEEDAFHFVSYVPVNGRLYELDGLREGPIDLGACNQDDWISAVRPVIEKR 207

************************************************************

His-TEV-UCH37 IQKYSEGE**I**RFNLMAIVSDRKMIYEQKIAELQRQLAEEEPMDTDQGNSMLSAIQSEVAKN 300

NS-UCH37 IQKYSEGE**I**RFNLMAIVSDRKMIYEQKIAELQRQLAEEEPMDTDQGNSMLSAIQSEVAKN 267

UCH37deltaCTD IQKYSEGE**I**RFNLMAIVSDRKMIYEQKIAE------------------------------ 237

******************************

His-TEV-UCH37 QMLIEEEVQKLKRYKIENIRRKHNYLPFIMELLKTLAEHQQLIPLVEKAKEKQNAKKAQE 360

NS-UCH37 QMLIEEEVQKLKRYKIENIRRKHNYLPFIMELLKTLAEHQQLIPLVEKAKEKQNAKKAQE 327

UCH37deltaCTD ------------------------------------------------------------ 237

His-TEV-UCH37 TK 362

NS-UCH37 TK 329

UCH37deltaCTD -- 237

**>His-TEV-UCH37**

MHHHHHHGKPIPNPLLGLDSTENLYFQGIDPFTMTGNAGEWCLMESDPGVFTELIKGFGCRGAQVE**E**I**W**SLEPENFEKLKPVHGLIFLFKWQPGEEPAGSVVQDSRLDTIFFAKQVINNA**C**ATQAIVSVLLNCTHQDVHLGETLSEFKEFSQSFDAAMKGLALSNSDVIRQVHNSFARQQMFEFDTKTSAKEEDAFHFVSYVPVNGRLYELDGLREGPIDLGACNQDDWISAVRPVIEKRIQKYSEGE**I**RFNLMAIVSDRKMIYEQKIAELQRQLAEEEPMDTDQGNSMLSAIQSEVAKNQMLIEEEVQKLKRYKIENIRRKHNYLPFIMELLKTLAEHQQLIPLVEKAKEKQNAKKAQETK-

**>NS-UCH37**

STGNAGEWCLMESDPGVFTELIKGFGCRGAQVE**E**I**W**SLEPENFEKLKPVHGLIFLFKWQPGEEPAGSVVQDSRLDTIFFAKQVINNA**C**ATQAIVSVLLNCTHQDVHLGETLSEFKEFSQSFDAAMKGLALSNSDVIRQVHNSFARQQMFEFDTKTSAKEEDAFHFVSYVPVNGRLYELDGLREGPIDLGACNQDDWISAVRPVIEKRIQKYSEGE**I**RFNLMAIVSDRKMIYEQKIAELQRQLAEEEPMDTDQGNSMLSAIQSEVAKNQMLIEEEVQKLKRYKIENIRRKHNYLPFIMELLKTLAEHQQLIPLVEKAKEKQNAKKAQETK*

**> UCH37deltaCTD**

MTGNAGEWCLMESDPGVFTELIKGFGCRGAQVE**E**I**W**SLEPENFEKLKPVHGLIFLFKWQPGEEPAGSVVQDSRLDTIFFAKQVINNA**C**ATQAIVSVLLNCTHQDVHLGETLSEFKEFSQSFDAAMKGLALSNSDVIRQVHNSFARQQMFEFDTKTSAKEEDAFHFVSYVPVNGRLYELDGLREGPIDLGACNQDDWISAVRPVIEKRIQKYSEGE**I**RFNLMAIVSDRKMIYEQKIAE
