# Supplementary material for: Branched ubiquitin chain binding and deubiquitination by UCH37 facilitate proteasome clearance of stress-induced inclusions
Source: eLife. 2021 Nov 11;10:e72798. doi: 10.7554/eLife.72798 (PMC8635973; doi:10.7554/eLife.72798)
Supplement: Transparent reporting form [file elife-72798-transrepform1.docx]

***eLife’s* transparent reporting form**

We encourage authors to provide detailed information *within their submission* to facilitate the interpretation and replication of experiments. Authors can upload supporting documentation to indicate the use of appropriate reporting guidelines for health-related research (see [EQUATOR Network](http://www.equator-network.org/%20)), life science research (see the [BioSharing Information Resource](https://biosharing.org/)), or the [ARRIVE guidelines](http://www.plosbiology.org/article/info:doi/10.1371/journal.pbio.1000412) for reporting work involving animal research. Where applicable, authors should refer to any relevant reporting standards documents in this form.

If you have any questions, please consult our Journal Policies and/or contact us: [editorial@elifesciences.org](mailto:editorial@elifesciences.org).

**Sample-size estimation**

- You should state whether an appropriate sample size was computed when the study was being designed
- You should state the statistical method of sample size computation and any required assumptions
- If no explicit power analysis was used, you should describe how you decided what sample (replicate) size (number) to use

Please outline where this information can be found within the submission (e.g., sections or figure legends), or explain why this information doesn’t apply to your submission:

Sample sizes are indicated in figure legends and details of statistical methods are stated in the Materials and Methods. Each sample size was decided based on prior experience with the assays as well as practical considerations and generally accepted standards in the field. No explicit power analysis was used as the results were relatively consistent between samples.

Specifically, in main figure 5B-D, to quantify the RPT6 foci numbers in each cell, more than 100 (n >100) cells were measured for each cell type. In main figure 6E, FRAP analysis of GFP-UCH37 after 0.2 M sucrose treatment were performed with at least 20 foci from 7 or 8 cells from each cell line.

**Replicates**

- You should report how often each experiment was performed
- You should include a definition of biological versus technical replication
- The data obtained should be provided and sufficient information should be provided to indicate the number of independent biological and/or technical replicates
- If you encountered any outliers, you should describe how these were handled
- Criteria for exclusion/inclusion of data should be clearly stated
- High-throughput sequence data should be uploaded before submission, with a private link for reviewers provided (these are available from both GEO and ArrayExpress)

Please outline where this information can be found within the submission (e.g., sections or figure legends), or explain why this information doesn’t apply to your submission:

Our study contains both biological (measurements of biologically distinct samples) and technical (repeated measurements run on same samples) replicates. For all quantified data, the details of number of replicates, analyses, and statistical tests used can be found in Materials and Methods and Figure Legends. Specifically,

Figure 1E, 2C, 5B, 5F, 6C: two biological replicates.

Figure 6F and Figure 6-figure supplement 1E: two biological replicates and three technical replicates.

Figure 6-figure supplement 1D: three technical replicates.

All gel-based experiments and MST titrations were done multiple times and representative results were shown.

**Statistical reporting**

- Statistical analysis methods should be described and justified
- Raw data should be presented in figures whenever informative to do so (typically when N per group is less than 10)
- For each experiment, you should identify the statistical tests used, exact values of N, definitions of center, methods of multiple test correction, and dispersion and precision measures (e.g., mean, median, SD, SEM, confidence intervals; and, for the major substantive results, a measure of effect size (e.g., Pearson's r, Cohen's d)
- Report exact p-values wherever possible alongside the summary statistics and 95% confidence intervals. These should be reported for all key questions and not only when the p-value is less than 0.05.

Please outline where this information can be found within the submission (e.g., sections or figure legends), or explain why this information doesn’t apply to your submission:

Details of analyses and statistical tests used can be found in Materials and Methods and Figures Legends. Specifically,

Figure 1E, 5F: mean values and standard deviations are plotted.

Figure 2A, Figure2-S1A, Figure 2C, Figure 6-S1D: best fit values are shown with standard errors.

Figure 6F, Figure6-S1E: mean values and Coefficient of Variations are plotted.

Figure 5B, 5F: unpaired t-tests were performed between each cell type and P: *, p<0.05, **, p<0.01, ***, p < 0.001; ****, p < 0.0001; n.s., not significant.

(For large datasets, or papers with a very large number of statistical tests, you may upload a single table file with tests, Ns, etc., with reference to sections in the manuscript.)

**Group allocation**

- Indicate how samples were allocated into experimental groups (in the case of clinical studies, please specify allocation to treatment method); if randomization was used, please also state if restricted randomization was applied
- Indicate if masking was used during group allocation, data collection and/or data analysis

Please outline where this information can be found within the submission (e.g., sections or figure legends), or explain why this information doesn’t apply to your submission:

N/A

**Additional data files (“source data”)**

- We encourage you to upload relevant additional data files, such as numerical data that are represented as a graph in a figure, or as a summary table
- Where provided, these should be in the most useful format, and they can be uploaded as “Source data” files linked to a main figure or table
- Include model definition files including the full list of parameters used
- Include code used for data analysis (e.g., R, MatLab)
- Avoid stating that data files are “available upon request”

Please indicate the figures or tables for which source data files have been provided:

Source data file is provided for quantitative mass spectrometry result (Figure 6F and Figure 6-figure supplement 1E).
